# Long-term trends in educational inequalities in alcohol-attributable mortality, and their impact on trends in educational inequalities in life expectancy

**DOI:** 10.3389/fpubh.2024.1355840

**Published:** 2024-12-18

**Authors:** Jesús-Daniel Zazueta-Borboa, Wanda M. J. Van Hemelrijck, Nicolás Zengarini, Alison Sizer, Anton E. Kunst, Pekka Martikainen, Fanny Janssen

**Affiliations:** ^1^Netherlands Interdisciplinary Demographic Institute-KNAW, The Hague, Netherlands; ^2^Faculty of Spatial Sciences, Population Research Centre, University of Groningen, Groningen, Netherlands; ^3^Epidemiology Unit, ASL TO3, Piedmont Region, Grugliasco, Italy; ^4^Department of Information Studies, University College London, London, United Kingdom; ^5^Department of Public Health, Amsterdam UMC, University of Amsterdam, Amsterdam, Netherlands; ^6^Population Research Unit, Faculty of Social Sciences, University of Helsinki, Helsinki, Finland; ^7^Max Planck Institute for Demographic Research, Rostock, Germany

**Keywords:** educational-inequalities, alcohol-attributable mortality, trends, Europe, life-expectancy

## Abstract

**Background:**

Previous studies on socio-economic inequalities in mortality have documented a substantial contribution of alcohol-attributable mortality (AAM) to these inequalities. However, little is known about the extent to which AAM has contributed to time trends in socio-economic inequalities in mortality.

**Objective:**

To study long-term trends in educational inequalities in AAM and assessed their impact on trends in educational inequalities in life expectancy in three European countries.

**Methods:**

We analyzed cause-specific mortality data by educational group (low, middle, high) for individuals aged 30 and older in England and Wales, Finland, and Turin (Italy) over the 1972–2017 period. To estimate AAM, we used the multiple causes of death approach for England and Wales and Finland (1987–2017), and a recently introduced method for Turin (Italy). We used segmented regression analysis to study changes in absolute educational inequalities in AAM, measured by the Slope Index of Inequality (SII). We assessed the contribution of AAM to trends in educational differences in remaining life expectancy at age 30 (e30) using cause-deleted life tables.

**Results:**

AAM increased more among the low-educated than the high-educated in England and Wales (1972–2017) and Finland (1987–2007). In contrast, in Finland (2007 onwards) and Turin (1972–2017), AAM decreased more among the low-educated than the high-educated. In England and Wales, AAM contributed 37% (males) and 24% (females) of the increase in educational inequalities in e30. In Finland in 1987–2007, AAM contributed 50% (males) and 34% (females) of the increase in educational inequalities in e30. AAM also contributed to recent trend breaks, such as to the onset of an increase in educational inequalities in e30 in England and Wales, and to the onset of a decline in educational inequalities in e30 in Finland after 2007.

**Discussion:**

AAM mortality contributed substantially not only to levels of, but also to changes in educational inequalities in e30 in the studied populations. Reducing the impact of alcohol on mortality among low-educated groups may positively affect trends in educational inequalities in life expectancy.

## 1 Introduction

Alcohol-attributable mortality (AAM) has been shown to be higher among people with low than with high socio-economic status (SES), regardless of whether SES is measured by education, occupation, or income (1, 2). These inequalities in AAM have persisted over time in Europe (3–9). Between 1980 and 2009, absolute educational inequalities in AAM even increased in northern European countries (Finland and Denmark), while they decreased in southern European cities (Turin [Italy] and Madrid [Spain]) (10). Trends in inequalities in AAM have likely contributed to trends in educational inequalities in all-cause mortality and life expectancy in Europe, given the evidence indicating that AAM and alcohol consumption greatly affect *levels* of socio-economic mortality inequalities across Europe (5, 11, 12), while AAM determines national life expectancy *trends* in Europe (13).

However, of the studies that have assessed the impact of AAM on educational inequalities in mortality (5–8, 14–20), only a few have focused on the impact of AAM on trends in educational inequalities in mortality (6, 20). Moreover, previous cross-national studies on SES inequalities in AAM foremost estimated AAM using underlying causes of death (2, 5) thereby counting purely the deaths from conditions that are considered wholly attributable to alcohol, such as alcohol poisoning and liver cirrhosis—even though this approach is known to result in a substantial underestimation of AAM (18, 21, 22) affecting the level of AAM across SES groups and therefore the level of SES inequalities in AAM (23). Thus, the contribution of AAM to levels of and trends in SES inequalities in mortality cannot be adequately measured using such estimates of AAM.

The few studies that used the multiple cause of death method to estimate AAM (6–8, 18), which is regarded as adequate (21), found that AAM contributed substantially to SES inequalities in mortality at specific points in time. For Sweden, the contribution of AAM to educational inequalities in temporary life expectancy 30–74 (the expected number of years lived between those ages) remained stable over the 1991–2008 period, at around 17% for males and 8% for females (7). Among Nordic countries (Sweden, Denmark, Finland, and Norway) AAM accounted for 13–36% (males) and 1–6.8% (females) of the income differences in temporary life expectancy 25–79 over the 1995–2007 period (8). In Spain, the contribution of AAM to educational inequalities in life expectancy at age 30 between 2016 and 2018 was estimated at around 3.2% for males and 0.7% for females (18). Only one previous study that estimated AAM using the multiple cause of death approach, assessed the contribution of AAM to *trends in* socio-economic mortality. In their analysis, Martikainen et al. (6) examined the effects of alcohol and smoking on trends in differences in remaining life expectancy at age 20 between the highest and lowest income quintiles between 1988 and 1992 and 2003–2007 in Finland. They found that in the absence of AAM, the increase in income differences in life expectancy would be 67% lower among males and 45% lower among females.

All in all, however, the impact of AAM on trends in educational inequalities in mortality remains to some extent patchy, and current comparative evidence on the long-term trends in educational inequalities in AAM is based on a suboptimal measurement of alcohol-attributable mortality. Therefore, our objective is to study the long-term trends in educational inequalities in AAM, and to assess the impact of AAM on trend changes in educational inequalities in life expectancy in three European countries.

We study England and Wales, Finland, and Turin (Italy), which have different drinking cultures and national alcohol policies. We use estimates of AAM that consider not only deaths from underlying causes of death wholly related to alcohol deaths, but also include an estimate of alcohol-related mortality from diseases partly related to alcohol. In addition, we use annual mortality data, which enables us to perform a detailed analysis of trends in socio-economic inequalities in AAM, including potential trend changes, and to estimate their effects on trends in educational inequalities in life expectancy.

## 2 Methods

### 2.1 Data

We used individually linked all-cause and cause-specific mortality data by educational attainment, sex, and five-year age groups (30–34, 35–39…0.95+) for England and Wales (Office for National Statistics Longitudinal Study) (24–26), Finland (Statistics Finland), and Turin (Italy) (Turin Longitudinal Study) (27) from the early 1970s onwards. These data stem from longitudinal follow-up designs in which individual data on mortality and person-years are linked to information on educational attainment from registers (Finland) or population censuses (Turin [Italy]) or census subsamples (England and Wales) at the beginning of either a five-year or a 10-year mortality follow-up. The Office for National Statistics Longitudinal Study contains linked census and life events data for a 1% sample of the population of England and Wales. See Supplementary material for more information. For England and Wales and Turin (Italy), we selected the data for the 1972–2017 period for comparative purposes. For Finland, we restricted the analysis to the 1987–2017 period, because the multiple causes of death data needed for our estimation of AAM were only available from 1987 onwards.

To operationalize socio-economic status, we used the highest educational attainment obtained. We classified people with low (ISCED 0–2), middle (ISCED 3–4), and high (ISCED 5–6) educational attainment according to the International Standard Classification of Education (ISCED) from 1997 (28).

### 2.2 Estimation of alcohol-attributable mortality

Our estimate of alcohol-attributable mortality by educational attainment considers not only deaths from underlying causes of death wholly due to alcohol, but also includes an estimate of alcohol-attributable mortality from causes of death partly due to alcohol. For Finland and England and Wales, we used the “multiple cause of death” method, which has been used as well in recent studies on inequalities in AAM (6–8, 12) and is considered the most accurate AAM estimation method when the cause-of-death registration is of high high-quality (21, 22). The method uses both underlying and contributory cause of death data from death certificates to include not only deaths that are fully due to harmful alcohol consumption (like was done before—see introduction) but also deaths for which harmful alcohol consumption was a contributory cause of death. Similar to Van Hemelrijck et al. (23) we classified deaths as alcohol-attributable if one of the following causes was listed as either the underlying cause of death or one of the first three contributory causes of death on a death certificate: mental and behavioral disorders due to alcohol use (F10, G31.2), alcoholic liver disease and cirrhosis (K70, K73, and K74), accidental poisoning by alcohol (X45), and alcoholic cardiomyopathy (I42.6). This selection of causes was based on the list published by Rehm et al. (29). We excluded, however, causes of death that are not relevant for those aged 30 and older (e.g., P04.3 Fetus and newborn affected by maternal use of alcohol, Q86.0 Fetal alcohol syndrome), and excluded a few rarely occurring causes of death that caused issues for our study of trends over time due to ICD revisions. Compared to Mackenbach et al. (5) we added G31.2, K73 and K74 to account for ICD revisions and cross-country coding differences over time (30, 31). Given that F10, K70, X45 and I42.6 together represent over 90% of the deaths considered wholly due to alcohol in Europe (5), we do not expect these slightly different selections of causes to importantly impact our results. We focused on the first three contributory causes of death, in line with Martikainen et al. (6, 18) for Finland, and because the first three contributory causes are the most frequently reported in England and Wales (see Supplementary material). For England and Wales, multiple causes-of-death data were missing for the years 1987 up to 1992. Thus, we applied interpolation of the death rates through a three-year moving average approach to estimate them for this period as well.

For Turin (Italy), the Turin Longitudinal Study did not contain long-term multiple causes of death data. To still be able to obtain an adequate and comparable estimate of AAM, we used the AAM estimates for Italy (Turin) from the recently proposed method by Van Hemelrijck et al. (23). This method accounts for the underestimation of previous AAM estimates that purely relied on causes of death wholly attributable mortality, and generated—for Finland—comparable estimates of AAM by educational level compared to the MCOD method (23). The method estimates alcohol-attributable mortality from causes of death partly due to alcohol by comparing—for the general population aged 30–64, by sex—age-standardized mortality from diseases “wholly” caused by alcohol (see the list of causes in the first paragraph of this section) with age-standardized mortality obtained using a population-attributable fraction (PAF) approach, thereby using the age-and sex-specific PAFs from the Global Burden of Disease study. Subsequently, the sex-specific ratio between the two is applied to age-, sex-, education-, and year-specific wholly alcohol-related deaths. The PAF estimates for ages 30–64 were chosen as the basis for the upward adjustment of wholly alcohol-related deaths by educational level, because (i) until age 65 AAM levels for MCOD and PAF proved to be similar in Finland, (ii) the age patterns of AAM estimates proved to the comparable between the wholly method, the MCOD method, and the PAF-based method for those ages, and (iii) PAF-based estimates for older ages are considered less reliable (22, 32). See Supplementary material for more information on this estimation method.

### 2.3 Analytical strategy

First, we analyzed trends in AAM by educational level (low, middle, high) by computing directly age-standardized AAM rates (30+) (SAAM) by country, sex, and educational attainment, using the 2013 revised European Standard Population (European Commission 2013) as our standard population. Subsequently, we applied Loess-smoothing with span 0.9 and degree 2 to smooth SAAM over time to visualize the trends.

Second, we analyzed trends in absolute educational inequalities in AAM by computing the yearly Slope Index of Inequality (SII). This measure can be interpreted as the rate differences in AAM between those with the lowest and those with the highest educational level (33). To obtain SII, we first estimated the Relative Inequality Index (RII) using a multiplicative Poisson model for each country by year and sex, adjusted for age groups (34). We subsequently obtained the SII using the following formula and information on the SAAM for the general population:


$$SII=\frac{2*\mathrm{SAAM}*\left(RII-1\right)}{\left(RII+1\right)}$$

We used segmented regression to fit the trends in absolute educational inequalities in AAM by country and sex, thereby identifying potential changes in the trend. We did so using the R package *Segmented* (35), which fit a linear model on our SII time series. Subsequently, using a likelihood ratio test with *p*-value 0.05, we assessed whether a model with a trend break provided a better fit for the SII trend than the linear model. We repeated this process for a model with two trend breaks versus one trend break, and proceeded until we no longer identified significant improvements in the model fit. As the starting values for the potential trend breaks, we used the outcome of different Davies tests. As a sensitivity analysis, we applied a similar segmented regression approach to the trends in relative educational inequalities in AAM by country and sex measured by the RII. The results of this analysis is discussed in the methodological considerations section.

Third, we assessed the impact of AAM on trends in educational inequalities in remaining life expectancy at age 30 (e30). We decided to use e30 as the final mortality outcome because age-standardized mortality is less likely to adequately reflect the contribution of AAM, given that AAM is particularly important at younger ages. To assess the impact of AAM, we compared the trends in educational inequalities in e30 with and without AAM. We measured educational inequalities in e30 by subtracting e30 for the low-educated from e30 for the high-educated. These e30 values were obtained by applying standard period life table techniques (36) to age-specific mortality rates by country, year, sex, and educational level. We applied cause-deleted life tables (37) to calculate the respective e30 values without AAM. We applied segmented regression to the obtained trends in educational inequalities in e30 with and without AAM. We subsequently quantified the absolute and the relative contribution of AAM to trends in educational inequalities in e30, in line with Martikainen et al. (6), by estimating the difference between the change over time in educational inequalities in e30 with AAM and without AAM.

## 3 Results

**Figure 1:**
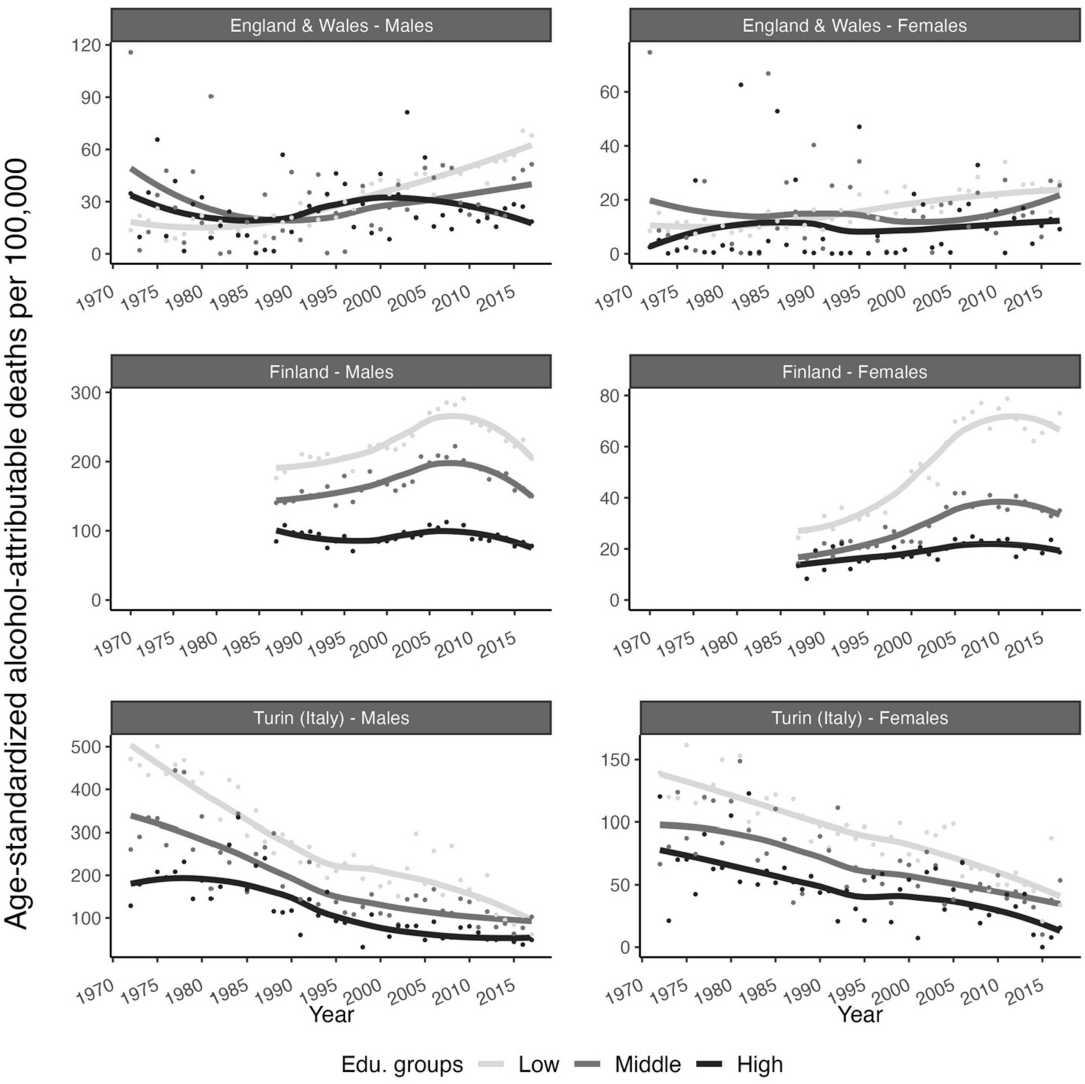
Smoothed time trends in age-standardized alcohol-attributable deaths per 100,000, by educational attainment level, sex, and country. England and Wales (1972–2017), Finland (1987–2017) and Turin (Italy) (1972–2017). European Standard Population 2013 as standard population. Point are the observed data while the lines are the smoothed trend from Loess-smoothing with span 0.9 and degree 2. Source data: ONS-Longitudinal Study, Statistics Finland, and Turin Longitudinal Study.

The findings indicated not only that levels of AAM were generally higher among the low-educated than the high-educated (except 1972–1990 in England and Wales), but also that trends in AAM differed between the low-educated and the high-educated, particularly in England and Wales (Figure 1). In England and Wales, the increases in AAM were larger among the low-educated than the high-educated; and among high-educated males, trends in AAM even decreased after approximately 2000. In Finland up to 2007–2010, AAM increased sharply among the low-educated, but fluctuated around low levels among high-educated males and increased only slightly among females. After 2007–2010 however, AAM declined more strongly among the low-educated than the high-educated. In Turin (Italy), AAM decreases were higher among the low-educated compared to the high-educated.

**Figure 2:**
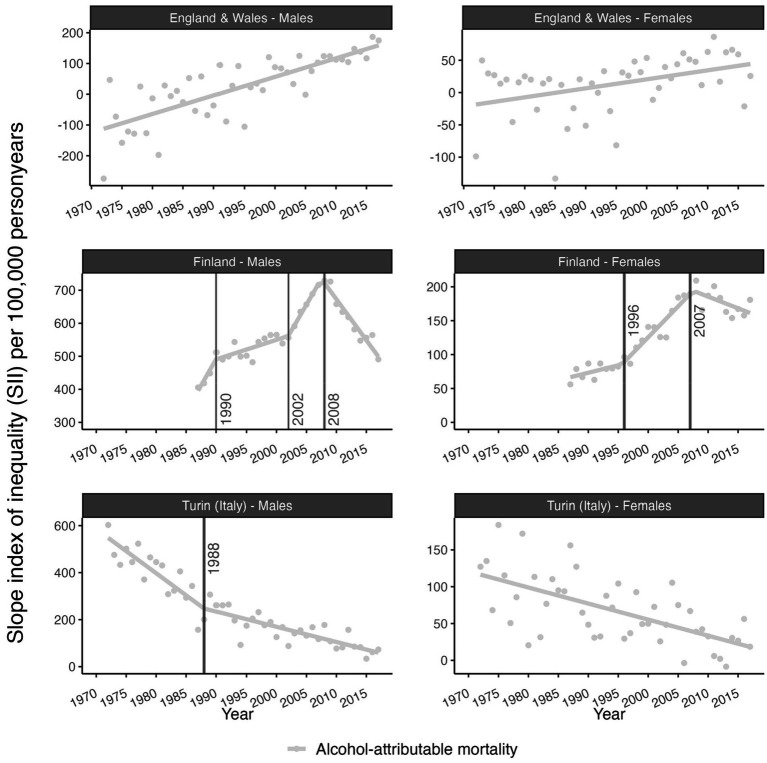
Time trends in absolute educational inequalities (SII) in alcohol-attributable mortality (AMM) for England and Wales (1972–2017), Finland (1987–2017), and Turin (Italy) (1972–2017). Points represent the observed values and lines represent the fitted trend obtained from segmented regression analysis (see Table A1). All trend breaks are statistically significant at *p*-value < 0.05. See Supplementary Table S1 for the *p*-values. Source data: ONS-Longitudinal Study, Statistics Finland, and Turin Longitudinal Study.

Consequently, absolute educational inequalities in AAM—as measured by the SII—increased in England and Wales and in Finland up to 2007 (females) and 2008 (males), whereas they decreased in Finland from 2007 (females) and 2008 (males) onwards and in Turin (Italy) throughout the study period (Figure 2). For both males and females in England and Wales and for females in Turin (Italy), our segmented regression analysis revealed no statistically significant trend breaks in the educational inequalities in AAM. For Finnish males, in addition to the reversal in 2008, we identified a change from a strong to a moderate increase in 1990, and from a moderate to a strong increase in 2002. For Finnish females, we found a change from a slow to a rapid increase in 1996 prior to the reversal in 2007. For males in Turin, we observed a change from a strong to a moderate decline in educational inequalities in AAM in 1988 (statistically significant). See Table A1 for the identified changes in the trend -break-years, slopes, and their CIs-, and see Supplementary Table S1 for their corresponding *p*-values.

**Figure 3:**
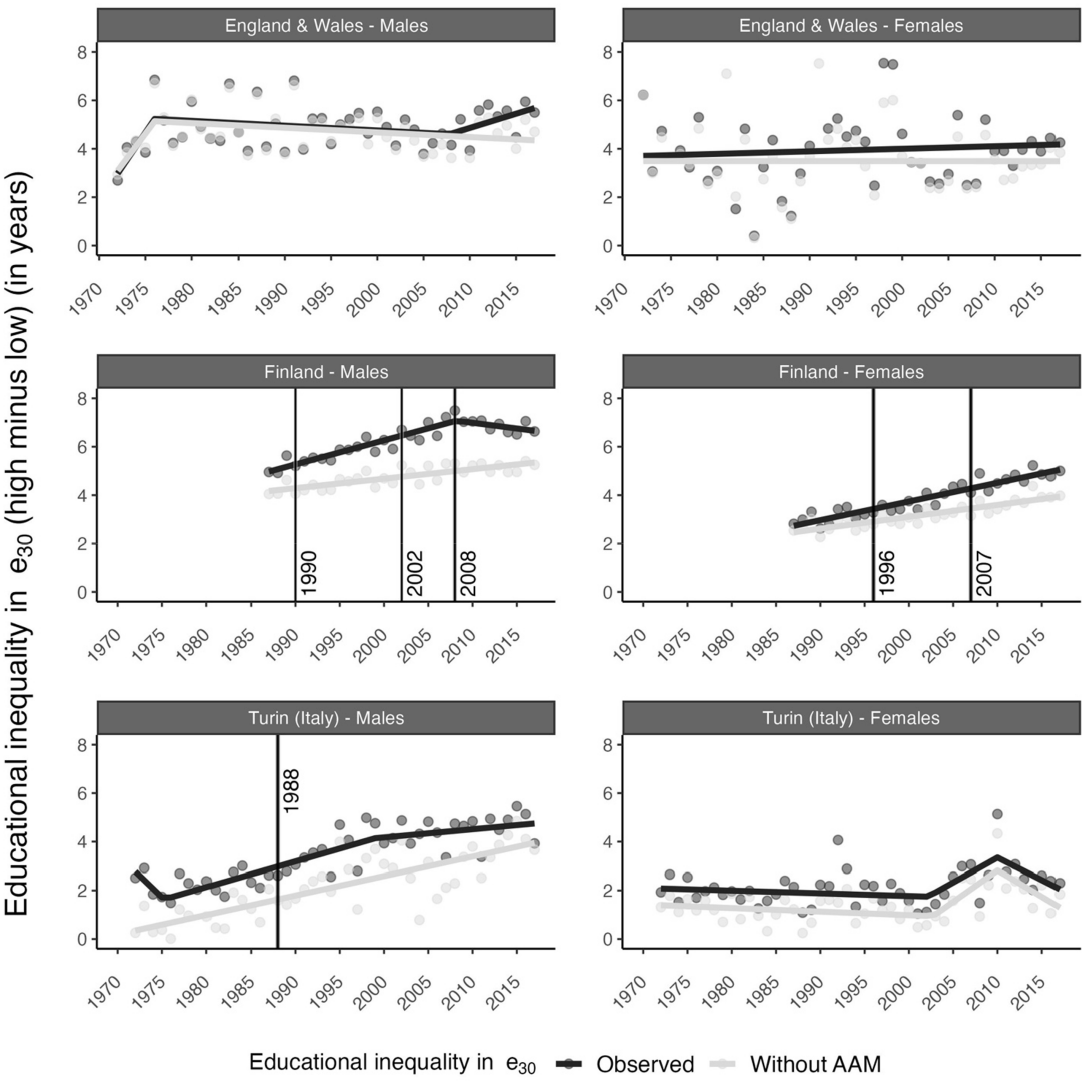
Time trends in educational inequalities in remaining life expectancy at age 30 (e30) with and without alcohol-attributable mortality (AAM) by sex and country. England and Wales (1972–2017), Finland (1987–2017), Turin (Italy) (1972–2017). Points represent the observed values and lines represent the fitted trends obtained from segmented regression analysis. All trend breaks are statistically significant at *p*-value < 0.05. See Supplementary Table 6 for the p-values. Vertical lines represent the trend breaks in absolute educational inequalities (SII) in AAM obtained from segmented regression analysis. Source data: ONS-Longitudinal Study, Statistics Finland, and Turin Longitudinal Study.

Overall, educational inequalities in e30 (e30 high-educated minus e30 low-educated) increased for British and Turin males (2017 versus 1972) and for Finnish males (2017 versus 1987); while for British and Italian females, these inequalities were roughly the same in 1972 and 2017, albeit with a small increase for British females from 1975 onwards (Figure 3). Without AAM, trends in educational inequalities in e30 generally followed the same overall trend, but at lower levels, particularly for Finnish and males in Turin. However, without AAM, the pace of increase was slower for British females (1975 onwards), Finnish males (1987–2008), and Finnish females (1987–2017). Moreover, for British, Finnish, and Turin males, the observed recent trend breaks in educational inequalities in e30 no longer appeared without AAM. The identified trend breaks in educational inequalities in AAM in Finland and in Turin (Italy) (see the vertical lines in Figure 3) did not coincide with a trend break in educational inequalities in e30, except among Finnish males in 2008.

Among British males, AAM contributed 37% of the overall increase (1972–2017) in educational inequalities in e30 (Table 1). Among British females, AAM contributed 24% of the increase in educational inequalities in e30 from 1980 onwards. Among Finnish males, AAM contributed 50% of the increase in inequalities in e30 in 1987–2008 and contributed 94% of the decline in inequalities from 2008 onwards. Among Finnish females, AAM contributed 34% of the increase in inequalities in e30 from 1987 to 2017. Among Turin males (1972–2017), educational inequalities in e30 would have increased by 3.4 years without AAM, compared to by 1.4 years with AAM, which implies a substantial negative contribution of AAM to the increase in inequalities. Among Turin females (1972–2017), educational inequalities in e30 increased by 0.37 years, and would have increased by 0.53 years without AAM.

**Table 1:** Change over time in educational inequalities in life expectancy at age 30 (e30) with and without alcohol-attributable mortality, and the contribution of alcohol-attributable mortality to the change over time in educational inequalities in e30.

Country	Sex	Period	Change over time in educational inequalities in e30 (in years)		Contribution of alcohol-attributable mortality to the change over time in educational inequalities in e30	
			Observed	Without alcohol-attributable mortality	Years	%
			(a)	(b)	(c)	(d)
England and Wales	Males	1972–2017	2.80	1.76	1.04	37.1
	Females	1980–2017	1.17	0.89	0.29	24.4
Finland	Males	1987–1990	0.25	0.01	0.24	95.6
		1990–2002	1.49	1.17	0.31	21.1
		2002–2008	0.80	0.08	0.73	91.1
		2008–2017	−0.86	−0.05	−0.81	94.0
		1987–2008	2.54	1.26	1.28	50.6
		1987–2017	1.68	1.21	0.47	28.0
	Females	1987–1996	0.47	0.26	0.21	45.0
		1996–2007	0.82	0.35	0.47	57.3
		2007–2017	0.90	0.83	0.07	7.6
		1987–2007	1.29	0.61	0.68	52.8
		1987–2017	2.18	1.44	0.75	34.3
Turin (Italy)	Males	1972–1988	0.09	1.34	−1.24	−1367.0
		1988–2017	1.32	2.07	−0.75	−57.0
		1972–2017	1.41	3.41	−2.00	−141.6
	Females	1972–2017	0.37	0.53	−0.16	−41.9

England and Wales (1972–2017), Finland (1987–2017), Turin (Italy) (1972–2017), by sex*. Note for Females in England and Wales (1980–2017) we used 1980 instead of 1975, since we observed a lot of fluctuation in the data between 1972 and 1979 at the beginning of the time series. All trend breaks are statistically significant at *p*-value < 0.05, and the years represent the trend breaks in absolute educational inequalities (SII) in AAM obtained from segmented regression analysis. See Supplementary Table S1 for the *p*-values. c = a-b. d = (a-b)/a * 100. Source data: ONS-Longitudinal Study, Statistics Finland, Turin Longitudinal Study.

## 4 Discussion

### 4.1 Summary of results

In our study, AAM increases were higher among the low-educated than the high-educated in England and Wales (1972–2017) and Finland (1987–2007 (females)/2008 (males)), which resulted in increasing absolute inequalities in AAM. In Finland (2007 (females) and 2008 (males), onwards) and Italy (1972–2017), AAM decreases were higher among the low-educated than the high-educated, which resulted in decreasing absolute inequalities in AAM.

AAM contributed 37% of the increase in educational inequalities in e30 among British males (1972–2017) and 24% of the increase among British females (1975–2017). AAM contributed 50% of the increase in inequalities in e30 among Finnish males (1987–2008) and 35% of the increase among Finnish females (1987–2017). For British, Finnish, and Italian males, the observed recent trend breaks of decreasing educational inequalities in e30 in Finland and the stagnation of increasing educational inequalities in e30 in Turin (Italy) were no longer found without AAM.

### 4.2 Interpretation of results

We observed that the changes in educational inequalities in AAM were heavily influenced by trends in AAM among the low-educated groups. Not only because the levels in age-standardized death rates by educational group was much higher among low-educated compared to high-educated, but also trends in age-standardized death in AAM differ between high and low-educated (Figure 1). This finding, together with the observation that the contribution of AAM to e30 was consistently higher among the low-educated than the high-educated (see Supplementary Figure S1), particularly in Finland and among British males, indicates the importance of AAM for trends in educational inequalities in e30.

For Finland, the observed trend changes in educational inequalities in AAM (Finnish males: 1990, 2002, and 2008; Finnish females: 1996 and 2007) may partly align with changes in economic circumstances and alcohol policies (see Supplementary Figure S3) that may have affected people with lower SES more than people with higher SES. The increases in educational inequalities in AAM among Finnish males and Finnish females partly coincided with the recession of the early 1990s and the introduction of new alcohol legislation in Finland in 1995, which, among other changes, liberalized access to medium strength beer and lifted the ban on public drinking (38). The observed acceleration of the increase in inequalities in AAM among Finnish males after 2004 took place during a period when taxes on alcohol—and, in turn, alcohol prices—were reduced (38). The subsequent decline in inequalities in AAM in Finland in 2008 occurred after an amendment to the alcohol act was introduced in January of that year, which included several tax increases in subsequent years (38). Previous research on the impact of these policies has suggested that the changes in the alcohol policies in 1995 and 2004 probably affected people with lower SES more than people with higher SES in absolute terms, but not necessarily in relative terms (4, 6, 17, 39–42). The observed changes in the contribution of AAM to trends in educational inequalities in e30 were also affected. These changes in economic circumstances and alcohol policies may have led to different trends by sex, by exacerbating pre-existing different drinking behaviors between males and females.

For Turin (Italy), AAM also contributed to levels of educational inequalities in e30, but less to the overall trends in educational inequalities in e30. The only exception was that for males the trend break in educational inequalities in e30 in the year 1999 no longer existed without AAM (Figure 3). The contribution of AAM to levels of educational inequalities in e30 in Turin (Italy) was linked to the high past levels of AAM, and the high levels of absolute inequality in AAM. These higher past levels of AAM in Italy—and in southern Europe in general—than in Finland and England and Wales—and north-western Europe in general—were observed in other studies as well (31, 43), and reflected the differences in recorded alcohol consumption (Supplementary Figure S3). This might be attributable to the Mediterranean drinking culture, which is characterized by the daily drinking of alcohol, mostly wine, and mostly during meals (30, 44, 45) which is an important difference in drinking behavior compared to north-western European countries. The differences in AAM by educational level we observed seemed to indicate that the Mediterranean drinking culture was more pronounced among the low-educated than the high-educated (46–48). However, between 1975 and 2010, the consumption of wine—and, in turn, overall alcohol consumption—has declined substantially in Italy (see Supplementary Figure S3), which has resulted in a decline in absolute educational inequalities in AAM (see Figure 2). Importantly, this decline in educational inequalities in AAM might have contributed to our finding that educational inequalities in e30 in Turin (Italy) did not further increase after 1999. Currently, absolute educational inequalities are lower in Italy than in Finland, possibly because binge drinking—which is highly socially stratified—is less common in southern Europe than in north-western Europe (5, 49). For Turin (Italy), in the Piemonte region -where Turin is located- the prevalence of binge drinking is similar to the level reported at the national level, which is generally lower than in north-western European countries (45).

For England and Wales, we observed gradually increasing inequalities in AAM, with AAM levels being relatively low. In western Europe, beer has long been the preferred beverage, which might have resulted in lower levels of alcohol-attributable mortality in western Europe than in southern Europe (30). However, over time, the importance of beer gradually declined (see Supplementary Figure S3), paving the way for other types of alcohol consumption that are potentially more socially patterned (5). Because of the generally low levels of AAM observed in England and Wales, AAM did not contribute much to the levels of inequalities in e30 in England and Wales. We found evidence that the increase in inequalities in AAM—including the recent reversal from declining to increasing educational inequalities in e30 among British men after 2008—disappeared. This reversal occurred in the context of a slowdown in life expectancy improvements in England and Wales that started in 2010 (50–52). According to previous research, this slowdown particularly affected neighborhoods with high levels of social deprivation (50), and has been linked to the introduction of strong austerity measures (51). It has been suggested that regional disparities in alcohol abuse among UK adults may partly explain the stagnation in life expectancy improvements in England and Wales since 2010 (52), and the concurrent increase in income inequalities in life expectancy in England and Wales (50) corroborates our findings.

### 4.3 Methodological considerations

The estimation of AAM is not straightforward, because of quality issues on the one hand, and data availability issues on the other hand (22). These issues are even more pertinent when measuring AAM by educational level (23). We decided to refrain from the AAM estimation method that merely considers the deaths from underlying causes of death wholly due to alcohol (“wholly method”), because it is known to result in a gross underestimation of AAM, both at the general level (21) and by educational level (12, 23). In addition, we decided against estimating AAM using a PAF-based approach, because estimates from the PAF approach at higher ages are considered less reliable (22, 32), and trends in AAM by educational attainment level using the PAF approach are considered less trustworthy compared to those obtained from the wholly-approach and the MCOD approach (23). Instead, we used multiple causes of death data for England and Wales and Finland, in line with recent research on socio-economic inequalities in AAM (6–8, 12). AAM estimates using multiple causes of death data are known to better reflect the true extent of alcohol-attributable mortality (22), and, in turn, its contribution to both the absolute levels and the trends in socio-economic inequalities in e30. For Turin (Italy),—the Turin Longitudinal Study does not contain long-term multiple causes of death data—and we applied the method recently proposed by Van Hemelrijck et al. (23) to be used in the many instances when MCOD by educational level are not readily available.

Although the applied methods are not similar across the countries, we regard it very likely that our estimates for Turin (Italy) are comparable to those for Finland and England and Wales. Firstly, AAM estimates from the method by Van Hemelrijck et al. proved largely similar to AAM estimates from the MCOD method for Finland (23). Secondly, for Turin(Italy) the obtained AAM levels for the general population aged 30–64 are—similar to Finland—equal to those AAM levels based on the PAF approach. Thirdly, for Turin(Italy), the obtained AAM levels for those aged 30 and over by educational level, fall—similar to Finland—in between the AAM estimates based on the wholly approach and based on the MCOD approach. Still however, some caution is required when comparing the obtained AAM estimates between Turin (Italy) on the one hand, and Finland and England and Wales, on the other hand. Related however to our main aim, own additional analysis revealed—as expected—substantially lower contributions of AAM to trends in educational inequalities in e30 when across the three countries the AAM estimates from the wholly approach were used compared to our approach (see Supplementary Table S5).

The linked cause-specific mortality data we used applied to the whole country for Finland, but to Turin city only for Italy. Meanwhile, for England and Wales, we relied on a 1% representative sample of the census populations. The small sample sizes for England and Wales and Italy (Turin), in combination with the many groups we distinguished (three educational attainment groups, two sexes, and 14 age groups), might call into question the robustness of our estimates and outcomes, especially given that alcohol-related mortality was not a very frequent event in those populations (5). Indeed, our trends in SAAM, in inequalities in AAM, and in inequalities in e30 fluctuated for these populations. We dealt with this issue by smoothing the trends, and by performing segmented regression to better capture the underlying trends. Nonetheless, the trends and trend breaks we identified for England and Wales and for Italy (Turin), particularly for women in the early years, should be interpreted with some caution.

Our findings for Turin (Italy) regarding long-term trends in educational inequalities in AAM and its impact on trends in educational inequalities in e30 cannot be directly translated to Italy as a whole. Although previous research has shown that levels and trends in life expectancy at age 30 (e30), and mortality for the total Turin population are similar to the ones observed in Italy (53) and that levels and trends in socio-economic inequalities in mortality in Turin largely resemble those of Italy as a country (54, 55), data on alcohol consumption, and associated morbidity and mortality likely differ by region in Italy (44, 45).

We focused on absolute inequalities instead of relative inequalities in this study, whereas most of the studies on trends in socio-economic inequalities in AAM (15) and all-cause mortality (56) reported both relative and absolute inequalities in mortality. Therefore, we also looked at trends in relative educational inequalities in AAM using the RII (see Supplementary Figure S2; Supplementary Tables S2–S4). This sensitivity analysis revealed different trends in relative inequalities in AAM than in absolute inequalities in AAM for British females and Turin females. However, our results regarding the contributions of AAM to trend changes in educational inequalities in e30 remained unaffected for Finnish males we observed a similar trend in relative and absolute educational inequalities in AAM, and for Finnish females, we did not identify a statistically significant trend break in relative educational inequalities in AAM; while for Turin males we observed the general tendency to decrease in educational inequalities in AAM.

## 5 Conclusion

Alcohol was found to be an important determinant not only of levels of (as demonstrated earlier in the literature), but also of trends and trend reversals in educational inequalities in e30 in the studied populations, albeit to different degrees. Particularly in Finland and among British males, alcohol contributed substantially to increased educational inequalities in e30 in specific periods. In addition, alcohol appears to have contributed to more recent changes. For example, alcohol might have played an important role in a trend break in educational inequalities in e30, since these inequalities were no longer observed for British, Finnish, and Turin males in the absence of AAM. Given that changes in educational inequalities in AAM are typically driven by AAM trends among low-educated groups, reducing the impact of alcohol on mortality among low-educated groups is essential to efforts to reduce educational inequalities in AAM and educational inequalities in life expectancy.

## Data Availability

The secondary (cause-specific) mortality data by educational level that we used as input for our analyses cannot be made publicly available, because the institutes that own the data (Statistics Finland, Office for National Statistics (ONS), and ASL TO3 (Epidemiology Unit)) apply a restricted access policy. Access to these data can, however, be requested through these institutes.

## Appendix

**Table A1:** Trend changes in absolute educational inequalities (SII) in alcohol-attributable-mortality (AAM), identified by the year of the trend breaks and the slopes of the different phases, including—in brackets—their confidence interval (95%) by sex and country.

Country	Sex	Break 1	Break 2	Break 3	Slope 1	Slope 2	Slope 3	Slope 4
England and Wales	Males	N.S.T.B.			6.03			
					(4.58, 7.48)			
	Females	N.S.T.B.			1.39			
					(0.47, 2.31)			
Finland	Males	1990	2002	2008	31.79	5.88	31.20	−24.75
		(1988–1991)	(2000–2004)	(2007–2009)	(12.55, 51.02)	(1.78, 9.98)	(20.92, 41.48)	(−29.48, −20.01)
	Females	1996	2007		2.88	9.66	−3.42	
		(1993–2000)	(2005–2008)		(−0.18, 5.96)	(7.00, 12.33)	(−6.49, −0.35)	
Turin (Italy)	Males	1988			−18.79	−6.47		
		(1983–1993)			(−23.67, −13.92)	(−8.65, −4.28)		
	Females	N.S.T.B.			−2.18			
					(−2.98, −1.38)			

England and Wales 1972–2017, Finland 1987–2017 and Turin (Italy) 1972–2017. All trend breaks are statistically significant at *p*-value < 0.05. See Supplementary Table S1 for the *p*-values. Source data: ONS-Longitudinal Study, Statistics Finland and Turin Longitudinal Study. N.S.T.B., No statistically significant trend break identified.
