# Supplementary material for: Long-term trends in educational inequalities in alcohol-attributable mortality, and their impact on trends in educational inequalities in life expectancy
Source: Front Public Health. 2024 Dec 18;12:1355840. doi: 10.3389/fpubh.2024.1355840 (PMC11691351; doi:10.3389/fpubh.2024.1355840)
Supplement: Supplementary file 1 [file Data_Sheet_1.PDF]

- Supplementary Material Tables and Figures

Long-term trends in educational inequalities in alcohol-attributable mortality, and their impact on trends in educational inequalities in life expectancy.

Supplementary Table 1. P-values from the likelihood ratio test in which the model with N breakpoints is compared to determine the statistical significance of trend breaks in absolute (SII) educational inequalities in mortality alcohol-attributable mortality, obtained from segmented regression analysis. England & Wales 1972-2017, Finland 1987-2017 & Turin (Italy) 1972-2017. *In bold values statistically significant at p-value <0.05. For England and Wales female, trend break 3, indicates statistical significance but was not included because the confidence interval for the years of breakpoints overlaps.*

Country	Sex	Break 1	Break 2	Break 3	Break 4	Break 5	Break 2 vs linear	Break 3 vs linear	Break 4 vs linear
England & Wales	Males	0.535	0.840	N.B.E.					
	Females	0.144	0.143	**0.019**	0.978	N.B.E.	0.101	**0.016**	
Finland	Males	0.000	0.000	0.003	0.239	N.B.E.	0.000	0.000	0.000
	Females	0.000	0.001	N.B.E.			0.000		
Turin (Italy)	Males	0.019	N.B.E.						
	Females	0.755	0.446	0.680	N.B.E.		0.703	0.815	

Source data: ONS-Longitudinal Study, Statistics Finland & Turin Longitudinal Study.
N.B.E.: No breakpoint was estimated.

frontiers

Supplementary Table 2. Trend changes in relative educational inequalities (RII) in alcohol-attributable-mortality (AAM), identified by the year of the trend breaks and the slopes of the different phases, including – in brackets – their confidence interval (95%) by sex and country. England and Wales 1972-2017, Finland 1987-2017 & Turin (Italy) 1972-2017. *All trend breaks are statistically significant at p-value <0.05. See Supplementary Table 1 for the p-values. See Supplementary Table 3 for the p-values.*

Country	Sex	Break 1	Break 2	Slope 1	Slope 2	Slope 3
England and Wales	Males	N.S.T.B.		0.05 (-0.06, 0.17)		
	Females	N.S.T.B.		-9.771 (-32.22, 12.67)		
Finland	Males	1993 (1989 – 1996)	2010 (2000 – 2019)	0.14 (0.04, 0.23)	0.01 (-0.01, 0.04)	-0.04 (-0.13, 0.05)
	Females	N.S.T.B.		0.06 (0.01 , 0.11)		
Turin (Italy)	Males	N.S.T.B.		-0.02 (-0.05, 0.02)		
	Females	N.S.T.B		0.04 (-0.03, 0.12)		

Source data: ONS-Longitudinal Study, Statistics Finland & Turin Longitudinal Study.

N.S.T.B.: No statistically significant trend break was estimated.

Supplementary Table 3. P-values from the likelihood ratio test in which the model with N breakpoints is compared to determine the statistical significance of trend breaks in relative (RII) educational inequalities in mortality alcohol-attributable mortality, obtained from segmented regression analysis. England & Wales 1972-2017, Finland 1987-2017, & Turin (Italy) 1972-2017. *In bold values statistically significant at p-value <0.05.*

Country	Sex	Break 1	Break 2	Break 3	Break 4	Break 2 vs linear	Break 3 vs linear
England & Wales	Males	0.3104	0.2834	N.B.E.			
	Females	0.1615	0.3437	N.B.E.			
Finland	Males	**0.0004**	0.2654	0.2494	N.B.E.	**0.00184**	**0.001622**
	Females	0.1364	0.0574	N.B.E.		**0.04578**	
Turin (Italy)	Males	0.1716	0.2289	N.B.E.		0.1664	
	Females	0.0950	0.5342	**0.0009**	N.B.E.	0.2020	**0.002671**

Source data: ONS-Longitudinal Study, Statistics Finland & Turin Longitudinal Study.
N.B.E.: No breakpoint was estimated.

Supplementary Table 4. Change over time in educational inequalities in life expectancy at age 30 (e30) with and without alcohol-attributable mortality, and the contribution of alcohol-attributable mortality to the change over time in educational inequalities in e30. England & Wales (1972-2017), Finland, 1987-2017, Turin (Italy) 1972-2018, by sex*. Analyzed periods selected based on trend breaks in relative (instead of absolute) inequalities in AAM.

Country	Sex	Period	Change over time in educational inequalities in e30 (in years)		Contribution of alcohol-attributable mortality to the change over time in educational inequalities in e30	
			All-cause mortality (a)	Without alcohol-attributable mortality (b)	Absolute c)	Relative d)
England & Wales	Males	1972-2017	2.80	1.76	1.04	37.0
	Females	1980-2017	1.17	0.89	0.29	24.4
Finland	Males	1987-1993	0.55	0.24	0.35	60.0
		1993-2010	1.54	0.66	0.76	53.0
		2010-2017	-0.42	-0.06	-0.27	183.0
		1987-2017	1.67	1.19	0.47	28.3
	Females	1987-2017	2.18	1.44	0.75	34.3
Turin (Italy)	Males	1972-2017	1.41	3.41	-1.01	-141.6
	Females	1972-2017	0.37	0.53	-0.16	-41.9

c = a-b

d = (a-b)/a * 100

Source data: ONS-Longitudinal Study, Statistics Finland, Turin Longitudinal Study.

Supplementary Table 5. Change over time in educational inequalities in life expectancy at age 30 (e30) with and without alcohol-attributable mortality using different alcohol-attributable mortality methods, and the contribution of alcohol-attributable mortality to the change over time in educational inequalities in e30. England & Wales (1972-2017), Finland, 1987-2017, Turin (Italy) 1972-2017, by sex*. Observed values, no Fitted values. *MCD = multiple cause of death approach, Wholly = wholly alcohol-attributable mortality.*

Country	Sex	Period	Change over time in educational inequalities in e30 (in years)			Relative contribution of alcohol-attributable mortality to the change over time in educational inequalities in e30 (%)	
			Observed	Without alcohol-attributable mortality using MCD	Without alcohol-attributable mortality using wholly	MCD	Wholly
			(a)	(b)	c)	d)	e)
England & Wales	Males	1972-2017	2.80	1.76	1.98	37.1	29.4
	Females	1980-2017	1.17	0.89	0.93	24.4	20.4
Finland	Males	1987-1990	0.25	0.01	0.28	95.6	-7.8
		1990-2002	1.49	1.17	1.41	21.1	5.5
		2002-2008	0.80	0.07	0.68	91.1	15.5
		2008-2017	-0.86	-0.05	-0.79	94.0	8.2
		1987-2008	2.54	1.26	2.36	50.6	7.3
		1987-2017	1.68	1.21	1.57	28.0	6.8
	Females	1987-1995	0.47	0.26	0.47	45.0	0.0
		1995-2007	0.82	0.35	0.69	57.3	14.9
		2007-2017	0.90	0.83	0.85	7.6	5.6
		1987-2007	1.29	0.61	1.16	52.8	9.4
		1987-2017	2.18	1.44	2.02	34.3	7.8
Turin (Italy)	Males	1972-1994	0.09	1.34	0.45	-1367.0	-293.4
		1994-2017	1.32	2.07	1.38	-57.0	-7.2
		1972-2017	1.41	3.41	1.82	-141.6	-25.7
	Females	1972-2017	0.37	0.53	0.42	-41.9	-14.3

c = a-b

d = (a-b)/a * 100

Source data: ONS-Longitudinal Study, Statistics Finland, Turin Longitudinal Study.

Supplementary Table 6. P-values from the likelihood ratio test in which the model with N breakpoints is compared to determine the statistical significance of trend breaks in remaining life expectancy at age 30 (e30) with and without alcohol-attributable mortality (AAM), obtained from segmented regression analysis. England & Wales 1972-2017, Finland 1987-2017, & Turin (Italy) 1972-2017. *In bold values statistically significant at p-value <0.05.*

Trend	Country	Sex	Break 1	Break 2	Break 3	Break 4	Break 5	Break 6	Break 2 vs linear	Break 3 vs linear	Break 4 vs linear	Break 5 vs linear
All Cause mortality	England & Wales	Males	0.0184	0.1085	N.B.E.				**0.014**			
		Females	0.4919	**0.0445**	0.3644	N.B.E.			0.106	0.124		
	Finland	Males	**0.0000**	0.1864	N.B.E.				**0.000**			
		Females	0.3712	0.2214	N.B.E.				0.288			
	Turin (Italy)	Males	0.1460	**0.0382**	0.2471	0.7587	N.B.E.		**0.035**	**0.040**	0.089	
		Females	0.2258	**0.0085**	0.0848	0.0721	0.3353	N.B.E.	**0.014**	**0.008**	**0.004**	**0.02462**
Without-AAM	England & Wales	Males	**0.0060**	0.1933	0.4275	N.B.E.			**0.009**	**0.019**		
		Females	0.2807	0.0617	N.B.E.				0.088			
	Finland	Males	0.2836	0.4705	N.B.E.				0.402			
		Females	0.1535	0.7931	N.B.E.				0.378			
	Turin (Italy)	Males	0.5521	**0.0021**	0.3352	N.B.E.			**0.009**	**0.015**		
		Females	0.0657	**0.0003**	0.9256	0.3640	N.B.E.		**0.000**	**0.001**	**0.003**	

Source data: ONS-Longitudinal Study, Statistics Finland, Turin Longitudinal Study.
N.B.E.: No breakpoint was estimated.

Supplementary Figure 1. Time trends in the contribution of alcohol-attributable mortality to levels in remaining life expectancy at age 30 (e30), by educational attainment groups, sex, and countries. England & Wales 1972-2017, Finland 1987-2017, Turin (Italy), 1972-2017.

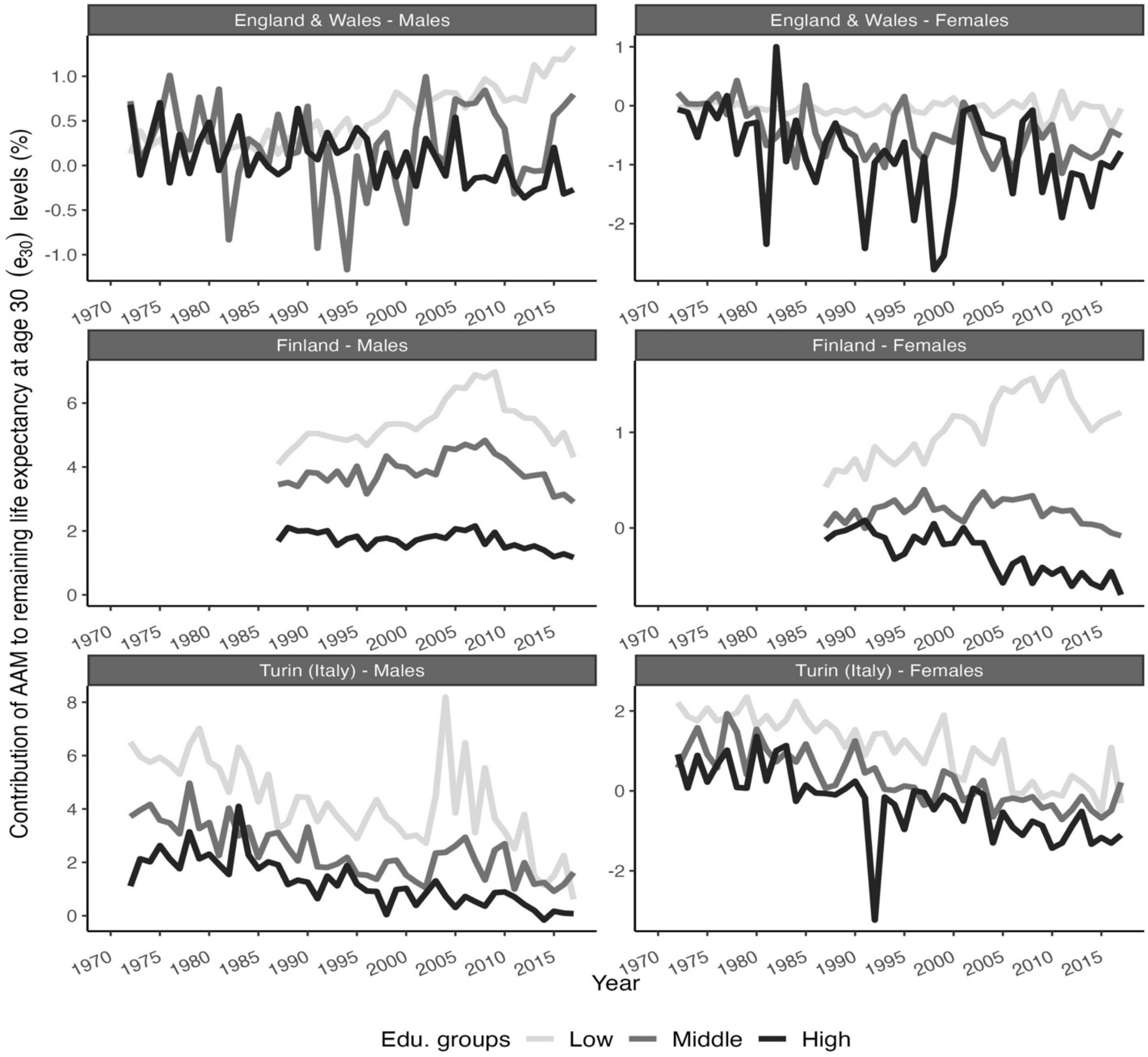

Source data: ONS-Longitudinal Study, Statistics Finland, and Turin Longitudinal Study.

Supplementary Figure 2. Time trends in relative educational inequalities (RII) in alcohol-attributable mortality (AAM) for England & Wales (1972-2017), Finland 1987-2017 and Turin (Italy) 1972-2017. Points represent observed values and lines represent the fitted trend obtained from segmented regression analysis.

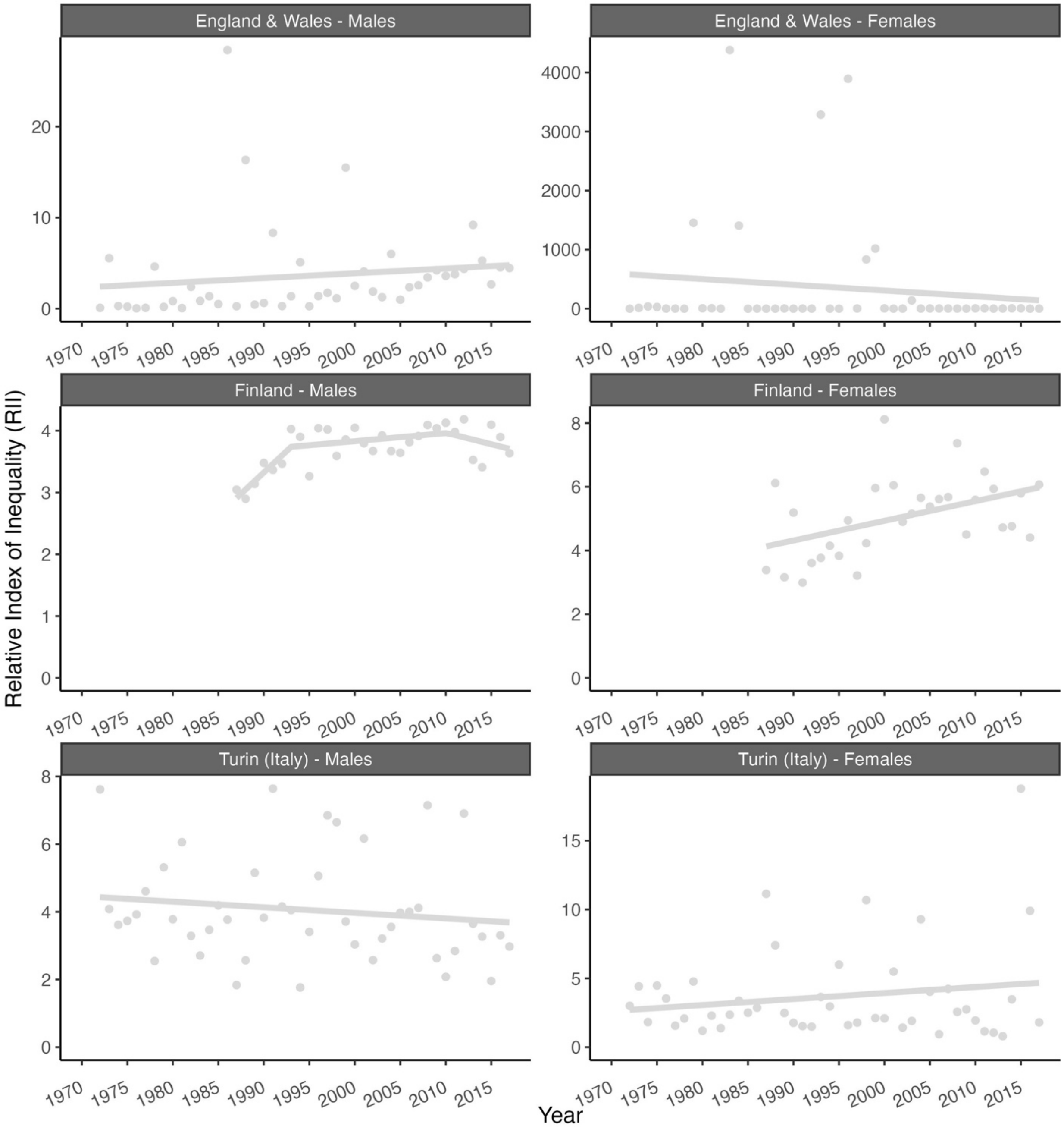

Source data: ONS-Longitudinal Study, Statistics Finland, and Turin Longitudinal Study.

Supplementary Figure 3. Recorded alcohol consumption (in liters of pure alcohol) per capita (15+), by country. Vertical dashed lines represent the introduction of national alcohol policies. *Note, for England and Wales we used the numbers for United Kingdom.*

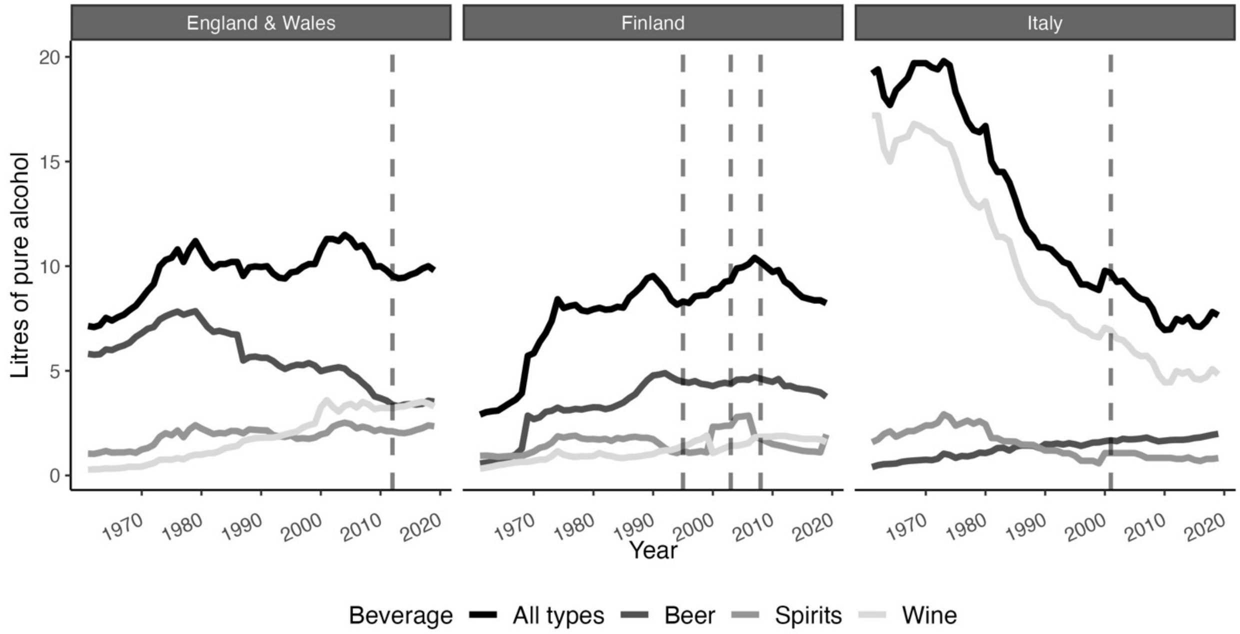

Data source: Global Information System on Alcohol and Health, last updated, 2022-06-13, last time consulted 2023-02-06.
