# Supplementary material for: Long-term trends in educational inequalities in alcohol-attributable mortality, and their impact on trends in educational inequalities in life expectancy
Source: Front Public Health. 2024 Dec 18;12:1355840. doi: 10.3389/fpubh.2024.1355840 (PMC11691351; doi:10.3389/fpubh.2024.1355840)
Supplement: Supplementary file 2 [file Data_Sheet_2.PDF]

Supplementary Material Detailed Data and Methods - Trend breaks in educational inequalities in alcohol-attributable mortality, and its contribution to trends in educational inequalities in life expectancy in selected European countries.

1- Detailed description of data

In the following link you can access a detailed description of the mortality data by educational level that we used.

https://www.futurelongevitybyeducation.com/background-information/ (Password = VICI_info).

2- Estimation of alcohol-attributable mortality for Italy

Estimates of alcohol-attributable mortality (AAM) based on causes of death that are wholly attributable to alcohol, such as alcohol poisoning and liver cirrhosis, are known to result in a substantial underestimation of AAM (Trias-Llimos et al., 2018; Trias-Llimos et al., 2022, Fihel, et al., 2023). Particularly when the aim is to assess the contribution of AAM to the levels of and the trends in educational inequalities in mortality – like in our study – using the abovementioned wholly AAM approach is not ideal. Instead, in our study, we relied on an estimate of AAM that used multiple causes of death data for England and Wales and Finland. However, for Italy, due to the lack of long-term multiple causes of death data by educational level, we used estimates from a recently introduced method (Van Hemelrijck et al. 2023) that adjusts upwards the case counts of wholly AAM to match the likely level of AAM, which for Italy was based on estimates of AAM for individuals aged 30-64 using the population-attributable fraction (PAF) approach that the Global Burden of Disease study employs. For Finland, the estimates from the PAF approach for individuals aged 30-64 were comparable to the AAM levels obtained using the multiple causes of death approach. Estimates from the PAF approach at higher ages are considered less reliable (Trias-Llimós et al., 2018; Marmet et al. 2016).

Please find below more information regarding the so-called enhanced-wholly approach that Van Hemelrijck et al (2023) developed. This information is copied directly from Van Hemelrijck et al. 2023, except for some small changes to the terminology used.

The adjusted-wholly method is based on an assessment of the pros and cons of different AAM estimation methods and the empirical comparison – for Finland – of trends, levels, and age distributions in AAM – both for the general population and by educational level – of three different AAM estimation methods: (1) the wholly cause of death approach (wholly_AAM), (2) the multiple causes of death approach (MCD_AAM), and (3) the population-attributable fraction (PAF_AAM) approach.

Three important observations form the basis of our novel estimation method. First, the levels in AAM are much lower for the wholly_AAM approach than for the multiple causes of death approach and the population-attributable fraction approach. Second, the trends in age-standardised AAM are largely identical for the wholly_AAM and the MCD_AAM approach, but are rather different for the PAF_AAM approach. Third, the age patterns in AAM are similar for the three

methods up to age 65, but become rather different (and unrealistic) for the PAF_AAM approach thereafter.

In line with these observations, our novel estimation method uses wholly_AAM as its basis, but corrects for known underestimations therein. More specifically, this novel method assumes a trend and age pattern similar to that of wholly_AAM, but adjusts the age-specific levels of AAM upwards so that they match the age-specific levels for the PAF_AAM for ages 30-64 (which, for Finland, resemble the age-specific levels for the MCD_AAM approach).

The data required to calculate the number of AAM deaths according to our new method consist of death counts for Italy by country, sex, educational level, and age according to both the wholly_AAM approach and the PAF_AAM approach.

The wholly AAM approach uses deaths from the following wholly alcohol-attributable causes of death: mental and behavioural disorders due to alcohol use (F10, G31.2); alcoholic liver disease and cirrhosis (K70, K73, and K74); accidental poisoning by alcohol (X45); and alcoholic cardiomyopathy (I42.6).

The PAF_AAM approach combines deaths from causes of death wholly attributable to alcohol (see the wholly AAM approach) with an estimate of alcohol-attributable mortality from causes of death partly attributable to alcohol, thereby using population-attributable fractions by country, year, sex, and age from the Global Burden of Disease study (GBD, 2017). See the table below for the 18 partly alcohol-attributable causes of death that were multiplied with their respective PAFs from the GBD.

Supplementary Table 1. List of causes of death and ICD 8-10 classification to adjust with partly alcohol-attributable mortality

Cause of death	ICD-8	ICD-9	ICD-10
Transport injuries	E800-E845	E800-E848	V01-V99, Y85
Self-harm and interpersonal violence	E950-E978, E990-E999	E950-E979, E990-E999	X60-X84, X85-Y09, Y35-Y36, Y87.0, Y87.1, Y89.0, Y89.1
Unintentional injuries	E850-E949 minus E860	E850-E949 minus E860	W00-X59 minus X45; Y40-Y84; Y88
Epilepsy	345	345	G40-G41
Cirrhosis and other chronic liver disease	570-573	570-573	K70-K76
Pancreatitis	577	577, 579.4	K85, K86
Tuberculosis	010-019	010-018, 137	A15-A19, B90

Lower respiratory infections	470-474, 480-483; 485-486, 466	480-487; 466	J10-J18 (influenza and pneumonia); J20-J22 (other acute lower respiratory infections)
Cardiovascular diseases	390-458	390-459	I00-I99
IHD	410-414	410-414	I20-I25
Stroke	430-438	430-438	I60-I69
Hypertensive heart disease/ hypertension	400-404	401-405	I10-I15
Atrial fibrillation and flutter (garbage code)	427.4	427.3	I48
Liver cancer	155, 197.8	155	C22
Larynx cancer	161	161	C32
Oesophageal cancer	150	150	C15
Breast cancer	174	174, 175	C50
Colon and rectum cancer	153-154	153-154	C18-C21
Cancer of lip, oral cavity, and pharynx	140-149	140-149	C00-C14

The process for obtaining the year-, sex-, education-, and age-specific adjusted-wholly_AAM counts consists of the following steps. First, we calculate directly age-standardised alcohol-attributable mortality rates according to both methods for the general population between ages 30 and 64, using the respective population counts from the European Standard Population revised in 2013 (SDR_AAM_30-64). Second, we divide the SDR_AAM_30-64 for the PAF_AAM approach by the SDR_AAM_30-64 for the wholly_AAM approach by year and sex for the years for which the GBD provides the PAFs (= 1990 up to 2017). Third, to identify trends similar to those observed using the wholly_AAM approach, we subsequently obtain a non-constant sex-specific adjustment factor by taking the average – by sex – of these yearly ratios. Fourth, we apply this non-constant sex-specific adjustment factor to the year, sex, education-, and age-specific wholly_AAM death counts for ages 30 and older.

References

Fihel, A., Trias-Llimós, S., Muszyńska-Spielauer, M. M., & Majerová, M. (2023). Alcohol-related mortality in four European countries: A multiple-cause-of-death study. *Drug and alcohol review*. https://doi.org/10.1111/dar.13624

Global Burden of Disease Study. (2017). GBD Results Tool. Retrieved July 26th, 2021, from https://gbd2017.healthdata.org/gbd-search/

Mackenbach JP, Kulhánová I, Bopp M, Borrell C, Deboosere P, Kovács K, Looman CW, Leinsalu M, Mäkelä P, Martikainen P, Menvielle G, Rodríguez-Sanz M, Rychtaříková J, de Gelder

R. (2015). Inequalities in Alcohol-Related Mortality in 17 European Countries: A Retrospective Analysis of Mortality Registers. PLoS Med, e1001909. doi: 10.1371/journal.pmed.1001909. PMID: 26625134; PMCID: PMC4666661.

Marmet, S., Rehm, J., & Gmel, G. (2016). The importance of age groups in estimates of alcohol-attributable mortality: impact on trends in Switzerland between 1997 and 2011. *Addiction*, *111*(2), 255-262.

Trias-Llimós, S., Martikainen, P., Mäkelä, P., & Janssen, F. (2018). Comparison of different approaches for estimating age-specific alcohol-attributable mortality: The cases of France and Finland. *PloS one*, *13*(3), e0194478.

Trias-Llimós S, Spijker JJA (2022). Educational differences in alcohol-related mortality and their impact on life expectancy and lifespan variation in Spain (2016-2018): a cross-sectional analysis using multiple causes of death. BMJ Open. 12(1):e053205. doi: 10.1136/bmjopen-2021-053205. PMID: 35074816; PMCID: PMC8788229.

Van Hemelrijck WMJ, Martikainen P, Zengarini N, Costa G, Janssen F (2023) The impact of estimation methods for alcohol-attributable mortality on long-term trends for the general population and by educational level in Finland and Italy (Turin). PLOS ONE 18(12): e0295760. https://doi.org/10.1371/journal.pone.0295760
